# Maize Kernel Broken Rate Prediction Using Machine Vision and Machine Learning Algorithms

**DOI:** 10.3390/foods13244044

**Published:** 2024-12-15

**Authors:** Chenlong Fan, Wenjing Wang, Tao Cui, Ying Liu, Mengmeng Qiao

**Affiliations:** 1 College of Mechanical and Electronic Engineering, Nanjing Forestry University, Nanjing 210037, China; fancl@njfu.edu.cn (C.F.); wangwenjin@njfu.edu.cn (W.W.); qiaomengmeng2024@njfu.edu.cn (M.Q.); 2 College of Engineering, China Agricultural University, Beijing 100083, China; cuitao@cau.edu.cn

**Keywords:** combine harvester, image processing, maize kernels, broken rate, detection

## Abstract

Rapid online detection of broken rate can effectively guide maize harvest with minimal damage to prevent kernel fungal damage. The broken rate prediction model based on machine vision and machine learning algorithms is proposed in this manuscript. A new dataset of high moisture content maize kernel phenotypic features was constructed by extracting seven features (geometric and shape features). Then, the regression model of the kernel (broken and unbroken) weight prediction and the classification model of kernel defect detection were established using the mainstream machine learning algorithm. In this way, the defect rapid identification and accurate weight prediction of broken kernels achieve the purpose of broken rate quantitative detection. The results prove that LGBM (light gradient boosting machine) and RF (random forest) algorithms were suitable for constructing weight prediction models of broken and unbroken kernels, respectively. The *r* values of the models built by the two algorithms were 0.985 and 0.910, respectively. SVM (support vector machine) algorithms perform well in constructing maize kernel classification models, with more than 95% classification accuracy. A strong linear relationship was observed between the predicted and actual broken rates. Therefore, this method could help to be an accurate, objective, efficient broken rate online detection method for maize harvest.

## 1. Introduction

**Figure 1:**
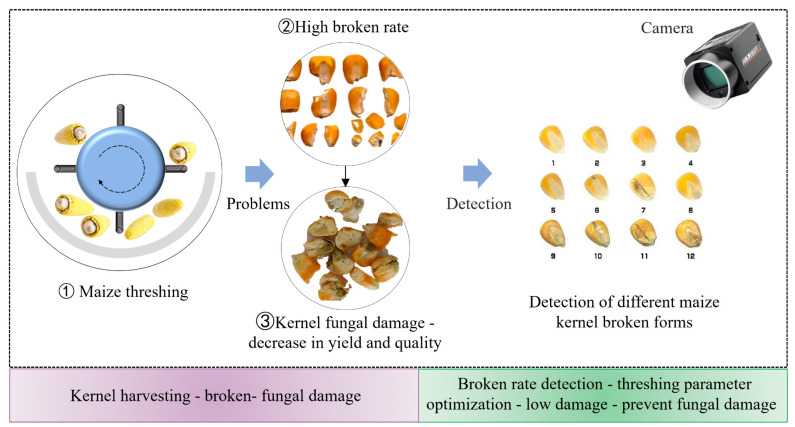
Low damage harvest and processing of corn kernels based on broken rate detection.

Maize is the largest grain crop in China, which is of great significance in ensuring food security [1]. Corn kernels are prone to breakage during mechanical harvesting, transportation, and processing. The breakage of corn kernels increases the risk of fungal damage, leading to a decline in both yield and quality [2,3]. The surface of the broken corn kernels is more susceptible to fungal damage. When the broken rate reaches 10%, the kernel mold rate will increase significantly, seriously affecting food safety and quality. Therefore, checking the broken rate in real time is necessary to adjust the operation parameters for maize harvesting. This can avoid excessive broken rate and reduce kernel mold risk (Figure 1). The detection of the broken rate of a traditional combine harvester during operation can only be observed manually after shutdown [4,5,6]. This method has a high misjudgment rate and low efficiency [7]. It is urgent to develop an automatic, non-destructive, and reliable technology for online detecting broken rates during maize harvesting [8]. With the wide application of machine vision and machine learning algorithms in the field of agricultural production, it can provide an effective online detection method for broken rates [9,10].

Machine vision and machine learning algorithms have been used to estimate the degree of grain breakage [11]. The kernel was divided into two categories: broken and unbroken kernel, and the image features of the maize kernel were processed using the machine learning method [12,13]. Kernel image features mainly include geometric features and shape features [14]. The geometric (area, perimeter, long axis, etc.) and shape features (circularity, rectangularity, etc.) are critical for recognizing maize broken and unbroken kernels [15]. Various phenotypic features of the maize kernel can be accurately identified by optimizing the classification algorithm of deep learning or machine learning [16]. Rasmussen et al. employed Faster R-CNN (regions with convolutional neural networks) to detect broken maize silage particles after harvesting; the results showed that the detection accuracy was 45.2%, which could identify large particles from miscellaneous ones [17]. Flores et al. adopted several CNN algorithms to learn and distinguish RGB and CIR images of corn and soybean, and it was found that Google Net is an effective discrimination method with an accuracy of 99.9% [18]. Li et al. used the yolov4-tiny model to train and recognize unbroken and broken maize kernel samples with a recognition accuracy of 93.0% [19]. Wu et al. compared the performance of a machine learning algorithm (SVM) and a deep learning algorithm (back-propagation neural networks algorithm) for maize kernel quality classification [20]. The results show that the support vector machine algorithm based on parameter optimization was superior to the back-propagation neural networks algorithm. Deep learning algorithms have achieved remarkable results in constructing classification models, but deep learning algorithm models usually require high-quality data for training. The model’s performance may deteriorate if the amount of data is insufficient or uneven. The machine learning algorithm can obtain better performance after fewer data requirements and shorter training time. It provides an essential basis for online rapid detection of grain quality.

Most current studies qualitatively estimate the degree of grain breakage by identifying the number of broken grains based on a machine learning algorithm. However, this method cannot achieve quantitative statistics on the broken rate [21,22] because the broken rate (%) is defined as the ratio of the broken kernel mass to all kernel mass in the sample. The above method cannot detect the broken kernel weight, which is an essential index for quantitatively calculating the broken rate. Therefore, accurately predicting the kernel mass is the key to quantitatively detecting the kernel broken rate.

Applying the machine learning regression algorithm provides an important technical means for rapidly predicting maize kernel weight [23]. Samrendra et al. proposed a stackable integrated model with rice digital fingerprints to predict the weight of rice and achieved high prediction accuracy [24]. The random forest (RF) model was employed to predict the individual pistachio kernel mass based on the features derived from a recursive image-processing algorithm [25]. Machine vision and machine algorithm-related technologies have also been applied to the weight detection of pistachios, carrots, and tomatoes, and good results have been achieved [26,27,28]. However, in the field of corn kernel broken rate detection, few researchers have quantified the broken rate by predicting kernel mass.

In summary, this article employs machine vision technology and machine learning algorithms to identify broken kernels and predict the ratio of broken kernel mass to the total kernel mass, achieving precise detection of corn broken rate. This paper used image processing technology to extract maize grains’ seven features (geometric features and shape features) as input. The kernel weight prediction and feature classification models were constructed using machine learning algorithms (SPR, LGBM, RF, SVM, KNN). The broken grain can be identified quickly, and the proportion of broken grain mass (broken rate) can be accurately predicted. At the same time, the influence weights of the seven kernel phenotypic features on the accuracy of broken grain recognition and prediction of grain weight were determined, which guided the further optimization of the online model of broken rate. The research results of this paper provide a theoretical reference for the online detection of maize grain surface features.

## 2. Materials and Methods

### 2.1. Maize Kernel Samples and Data Collection

**Figure 2:**
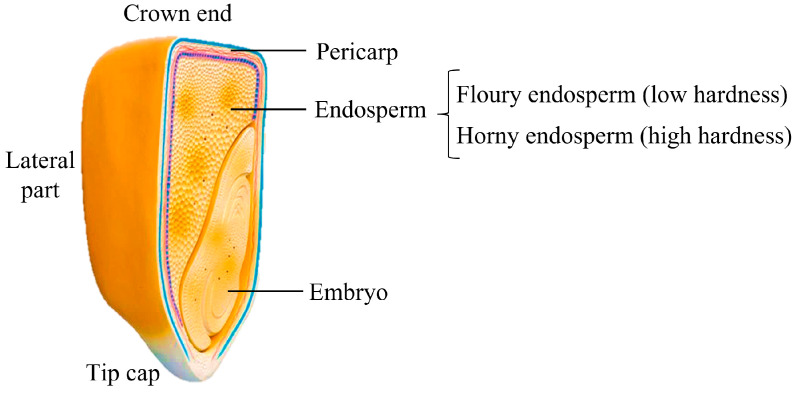
The structure of a corn kernel.

The dataset construction experiment of maize kernel was carried out in Nanjing, Jiangsu Province, in July 2023. The experimental material was Yichen 369 maize kernel, and its water content was 23.6%. A portable computer moisture analyzer (Kett, PM8188A, Osaka, Japan) was used to detect the moisture content of corn kernels. Place the corn kernels into the measuring chamber of the moisture analyzer (no manual weighing is required; it can automatically measure the sample mass). Close the lid to ensure a good seal. Start the moisture meter and wait 3 to 5 s to check the moisture content. Then, quickly remove the sample to conduct further testing. The structure of corn grains is shown in Figure 2.

In order to improve the accuracy of the detection model, the maize kernel sample set was divided into an unbroken kernel set and a broken kernel set to extract features, respectively. The broken kernel set covers all kinds of maize kernel broken forms, such as broken and cracked. In addition, maize kernel weight is a necessary index for calculating the broken rate. It is essential to label the weight of each kernel to train the data set to construct a predictive model of maize kernel weight. In the experiment, the weight of each maize kernel was marked by an electronic balance (Leqi, Suzhou, Jiangsu, China; the measurement range is 600 g, accuracy is 0.01 g). This process was repeated three times to calculate average values. The experimental results provide the basis for constructing the mapping model of maize kernel shape and weight prediction.

### 2.2. Design of Kernel Image Acquisition System

**Figure 3:**
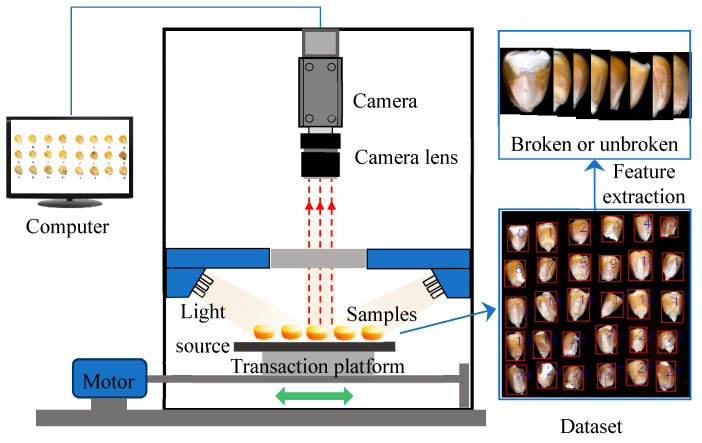
The working principle of maize broken rate detection system.

Kernel image acquisition is essential for extracting maize phenotypic features to construct the broken rate prediction model. The maize kernel image acquisition system mainly consists of the camera (Daheng, China, MER-531-20GC-P, 29 × 29 × 38.3 mm), camera lens (Computar FA, China, M2514-SW 2/3 “, 25 mm), light source (YISUDA, China, RH-RL9040-90 W), mobile platform, motor, and computer (Figure 3). The industrial camera selected for this study (resolution: 2592×2048; frame rate: 20 fps) can continuously collect images for the real-time detection of broken rates. Annular light sources were used for sample illumination to ensure that the system received even light. To obtain a complete image of the corn kernels, the camera distance was set at 0.20 m, resulting in a corn kernel image size of 2592 × 2048 pixels. To ensure clear images of the corn kernels, the camera needed to be calibrated before formal data collection. The camera was fixed on a stand, ensuring even illumination from the light source. A standard calibration target was then placed in the shooting position. The calibration target was positioned at the nearest focusing distance, the focus was adjusted, and a calibration image was captured. The clarity was checked to confirm there was no distortion. Finally, the camera settings were recorded for future reference.

The experimental process is as follows: 36 kernel images were collected each time. Then, the image was cut to extract the characteristic information of the kernel (broken or unbroken) according to the size of each kernel. Finally, area, perimeter, aspect ratio, long axis, short axis, circularity, and rectangularity were input features. The model between phenotypic feature and weight was constructed to predict kernel weight and to calculate the broken rate. Image processing and machine learning algorithms were performed using Python 3.7 through the open computer vision library OpenCV 3.4.3, the machine learning toolbox scikit-learn 0.24.2, and the deep learning toolbox Keras 2.4.3 [9].

### 2.3. Feature Extraction

The selection and extraction of maize kernel characteristic parameters directly affect the recognition accuracy of the maize broken kernels. Image features include color, geometric, shape, and texture features. Because the damaged part of the maize kernel is mainly concentrated in the embryo, the color feature of the damaged part is not obvious compared with the whole maize kernel. Therefore, the color feature of the maize kernel was not used in the manuscript; it only extracted the geometric and shape features as the detection index of the maize broken kernel [29]. Firstly, the maize kernel image was obtained by the broken rate detection system, and the image threshold was determined by the subtraction method to complete the maize kernel image segmentation. Secondly, the image boundary was marked, and the gray-level threshold method was used to segment the maize kernel image. Finally, the kernel weight was predicted, and the broken rate was calculated according to the maize kernel’s morphological feature.

**Figure 4:**
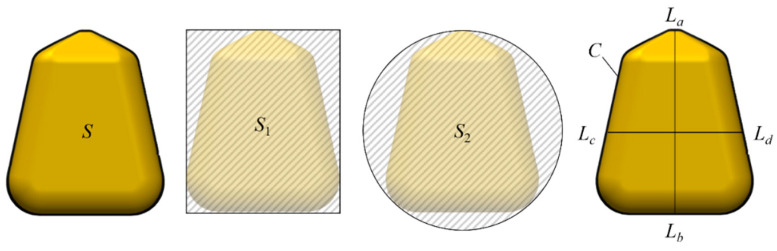
Schematic diagram of maize kernel geometry and appearance. S: kernel area; S_1_: minimum circumscribed rectangle area; S_2_: minimum circumscribed circle area; C: kernel perimeter; L_ab_: long axis of the kernel (a and b represent the endpoints of the longest axis of the kernel); L_cd_: short axis of the kernel (c and d represent the endpoints of the shortest axis.); e: circularity; R_r_: rectangularity.

Seven two-dimensional features of maize kernel were extracted in the manuscript: area (S), perimeter (C), aspect ratio (R_a_), long axis (L_ab_), short axis (L_cd_), circularity (e), and rectangularity (R_r_), as shown in Table 1. S, S_1_, S_2_, C, L_ab_, and L_cd_ represent kernel area, minimum circumscribed rectangle area, minimum circumscribed circle area, perimeter, long axis length, and short axis length (Figure 4). The long axis (L_ab_) refers to the straight-line distance between the two farthest points in the two-dimensional projection of a corn kernel, representing the maximum length of the kernel and typically aligned with the kernel’s main axis. L_cd_ is the line that passes through the centroid of the corn kernel and is perpendicular to the long axis (L_ab_). In the experiment, a total of 500 images were captured for both unbroken corn kernels and broken corn kernels. To construct a lightweight model while considering the characteristics of the kernels, 180 images (calibration set *n* = 135; prediction set *n* = 45) of broken kernels and 120 images (calibration set *n* = 90; prediction set *n* = 30) of unbroken kernels were randomly selected as the training sample set for the model. The reason for splitting the dataset into two subsets is that in small datasets, the additional split might lead to a smaller training set, which may be exposed to overfitting. To provide enough data for training, the validation set was used to assess the performance of the models [30]. Seven feature components were extracted as inputs. The kernel weight prediction model and the broken kernel classification model were constructed using the machine learning algorithm. Then, the ratio of broken kernel weight to total kernel weight (broken rate) was calculated.

### 2.4. Machine Learning Algorithm for Weight Prediction and Feature Classified Model Construction

**Figure 5:**
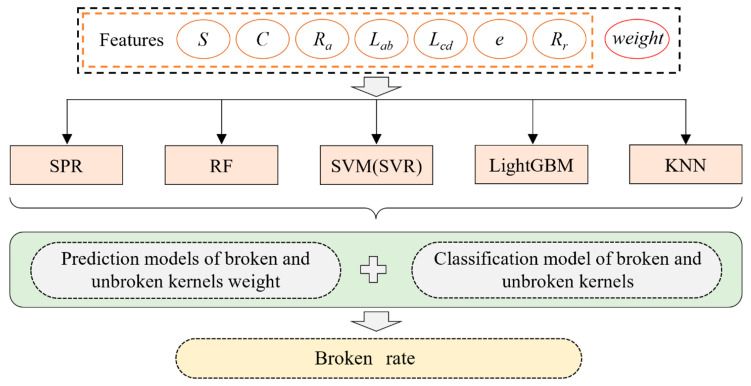
Prediction principle of broken rate based on machine learning algorithms.

The relationship between seven external characteristics and the weight of maize kernels was investigated by a machine learning algorithm in this manuscript (Figure 5). The kernel weight prediction model and the broken kernel classification model were constructed to provide the critical basis for calculating the broken rate. However, maize kernels of different weights have different features, and a simple linear algorithm may not be enough to describe the relationship between multiple features and kernel weight. This study selected linear and nonlinear machine learning algorithms to construct the high-precision broken rate model for maize kernels. Therefore, five machine learning algorithms, including stepwise polynomial regression (SPR), random forest (RF), support vector regression (SVR), and light gradient boosting machine (LightGBM), were used to build the weight prediction model for maize kernels.

Recognizing broken and unbroken kernels is the basis of calculating the broken rate. Because LGBM, RF, SVM, and KNN algorithms have both regression and classification functions, the above algorithm has been widely used in classifying and recognizing agricultural crops. Therefore, broken and unbroken kernels were classified by LGBM, RF, SVM, and KNN algorithms in this manuscript.

(1) Stepwise polynomial regression (SPR)

Area, perimeter, aspect ratio, et al. were used to predict maize kernel weight, but not all features had a significant relationship with grain weight. The SPR algorithm can identify the factors that significantly influence kernel weight prediction [31]. According to these features, the kernel weight prediction model was constructed to obtain higher prediction accuracy and improve the accuracy of the broken rate. This study’s linear regression model was built based on the SPR algorithm.

(2) Random forest (RF)

The random forest algorithm is subordinate to the bagging algorithm in ensemble learning, and a considerable forest model is constructed by several independent decision tree classifiers. The RF algorithm can introduce a double randomness factor to improve the algorithm’s anti-noise ability effectively [32]. Suppress the number of overfitting problems during data training. The adaptability of the algorithm to different data samples is strengthened. This study used Classification and Regression Trees to train the model.

(3) Support vector machine (SVM)

SVR is a regression analysis method based on support vector machines (SVM), which can map low-dimensional nonlinear input variables to high-dimensional linear spaces [33]. Then, linear regression is formed by finding the optimal interface. The error of all training samples obtained from the optimal interface is minimal. The choice of kernel function and corresponding parameters (penalty coefficient C, gamma) directly affects SVR’s learning and generalization ability. Therefore, in this study, the kernel functions were set as radial basis function (RBF), polynomial function (ploy), and sigmoid function (sigmoid). The corresponding formulas are (1), (2), and (3), respectively.

(1) $$K_{rbf}[X(i),X(i^{\prime })]=\exp(-\left\|X(i)-X(i^{\prime })\right\|/2\sigma^{2})$$

(2) $$K_{poly}[X(i),X(i^{\prime })]=[X(i{)}^{T}\cdot X(i^{\prime }){]}^{d}$$

(3) $$K_{sigmoid}[X(i),X(i^{\prime })]=\tanh(\lambda X(i{)}^{T}X(i^{\prime })+\xi )$$

where *d* is the number of polynomial degrees, *σ* and *λ* are the kernel parameters, *ξ* is constant, *i* = 1,2 ...81, *i*’= 1,2 ... 8l, *i* ≠ *i*’.

(4) Light Gradient Boosting Machine (LightGBM)

LightGBM regression is a machine learning algorithm based on a gradient enhancement framework [34]. LightGBM mainly uses a decision tree algorithm based on histograms to discretize floating point eigenvalues into k integers. Then, the histogram of width k is constructed. The histogram accumulates statistics when traversing the data. The optimal segmentation points are found according to the discrete values of the histogram. The core idea of the regression algorithm is to reduce the residual as the objective function and iteratively train the weak classifier. Finally, weak classifiers are linearly combined through an addition model to perform data classification or regression algorithms.

(5) K-nearest neighbors (KNN)

K-nearest neighbors (KNN) is a non-parametric and instance-based learning algorithm used in machine learning classification and regression tasks [35]. Given a dataset with labeled instances, KNN works by calculating the distance between the input sample and all other samples in the dataset. The k-nearest neighbors are then selected based on their distance from the input sample.

(4) $$\bar{y}=\frac{1}{K}\sum_{i=1}^{K}y_{i}$$

where *k* is the number of samples, *y_i_* and ȳ are sample target values and average values, respectively.

In this study, the hyperparameters of the LightGBM algorithm were set as follows: n_jobs = −1; colsample_bytree = 0.1 to 0.6 (step size: 0.1); n_estimators = 400 to 1000 (step size: 10); random_state = 2018. Set the kernel function types of the SVM algorithm as linear, polynomial, RBF, and sigmoid kernel; the values of penalty coefficient C were 0.1, 1, and 10, and the values of parameter gamma were 0.01, 0.1, and 1. The number of decision trees of the RF algorithm was 400~500 (preliminary screening), and the step size was 5; the maximum depth was 1~91 (step size: 0.1). The maximum features were 1~6. Set hyperparameters of the KNN algorithm: n_neighbors = 1 to 6 (step size: 1); weights = ‘distance’ or ‘uniform’; p = 1 to 6 (step size: 1). The RF, SVM, LGBM, and KNN algorithms with different combinations of hyperparameters are all used to build models from the training data. Five-fold cross-validation was used to initially screen models with lower errors [36].

### 2.5. Evaluation Metric

The experimental data were analyzed using the machine learning algorithm. Different algorithms were used to evaluate the accuracy of the kernel weight prediction model and broken kernel detection model, which provided the theoretical basis for online prediction of broken rate [37]. Among them, the model accuracy evaluation indexes include correlation coefficient (*r*), standard deviation (*SD*), and root-mean-square error (*RMSE*). The corresponding formulas are (5)–(7), respectively [38].

(5) $$r=\sqrt{1-\frac{\sum_{i=1}^{n}(y_{i,actual}-y_{i,predicted}{)}^{2}}{\sum_{i=1}^{n}(y_{i,actual}-y_{average}{)}^{2}}}$$

(6) $$SD=\sqrt{\frac{1}{N}\sum_{I=1}^{N}{\left(X_{i}-\bar{X}\right)}^{2}}$$

(7) $$RMSE=\sqrt{\frac{\sum_{i=1}^{n}(y_{i,actual}-y_{i,predicted}{)}^{2}}{N-1}}$$

### 2.6. Validation Test of Broken Rate Detection Model

**Figure 6:**
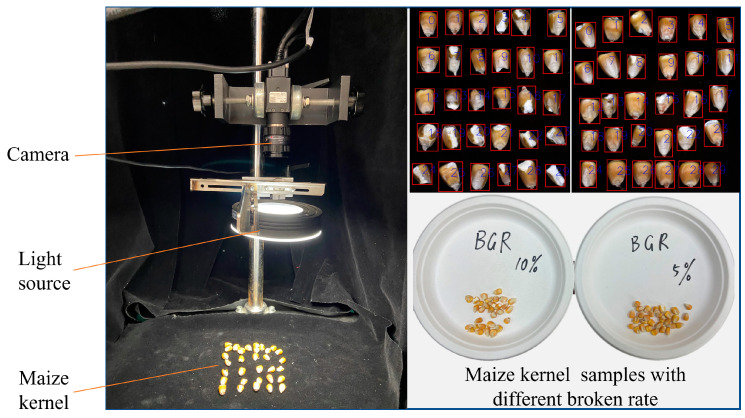
The performance verification test of the broken rate model.

The bench test was conducted to verify the performance of the broken rate detection model (Figure 6). The bench test focused on the online detection of maize kernels with different broken rates, analyzing the error level between the actual broken rate and the model-detected broken rate; 10 g of corn kernels (including broken and unbroken maize kernels) were randomly selected and arranged with different levels of broken rates (1% to 15%). Finally, the maize kernels were detected online using the constructed maize kernel breakage rate detection system. This process was repeated three times to take the average value. The formula for calculating the broken rate is shown in Equation (8).

(8) $$Z_{s}=\frac{m_{s}}{M_{i}}\times 100\%$$

where *m_s_* is the weight of broken grains in the sample, g; *M_i_* is the total weight of sample grains, g.

## 3. Results

### 3.1. Correlation Analysis of Maize Kernel External Feature and Weight

#### 3.1.1. Correlation Analysis of Broken Kernel

**Figure 7:**
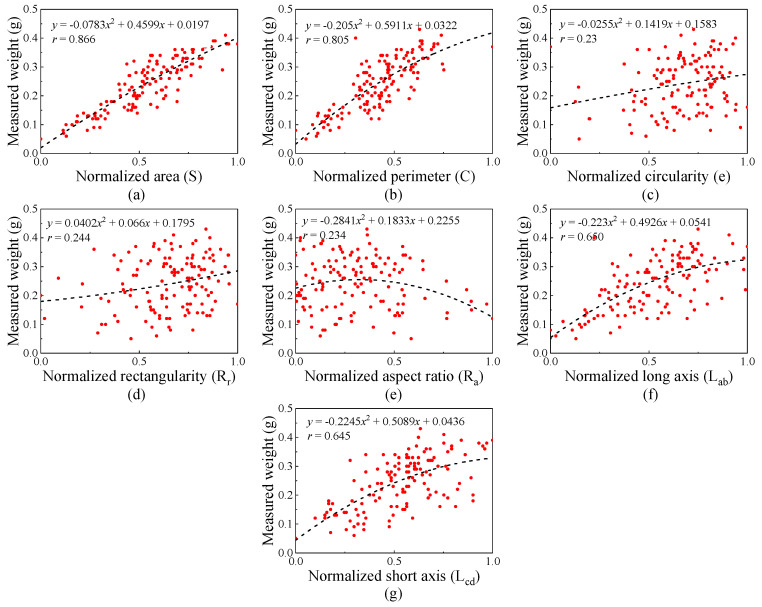
Correlation analysis between feature and weight for the broken kernel.

The quadratic polynomial was adopted to illustrate the correlation between individual features and the weight of broken kernel [9], which was ranked as area (*S*) > perimeter (*C*) > long axis (*L_ab_*) > short axis (*L_cd_*) > rectangularity (*R_r_*) > aspect ratio (*R_a_*) > circularity (*e*), as shown in Figure 7. The correlation between *S* and broken kernel weight was the highest, and *r* was higher than 0.85. The correlation between perimeter and weight (*r* = 0.805) was second only to that between area and weight. Because the projected area was more closely related to the volume of the broken kernel, the relationship between perimeter and volume was second. The *r* values of the long axis, short axis, rectangularity, aspect ratio, and circularity were low.

#### 3.1.2. Correlation Analysis of Unbroken Kernel

**Figure 8:**
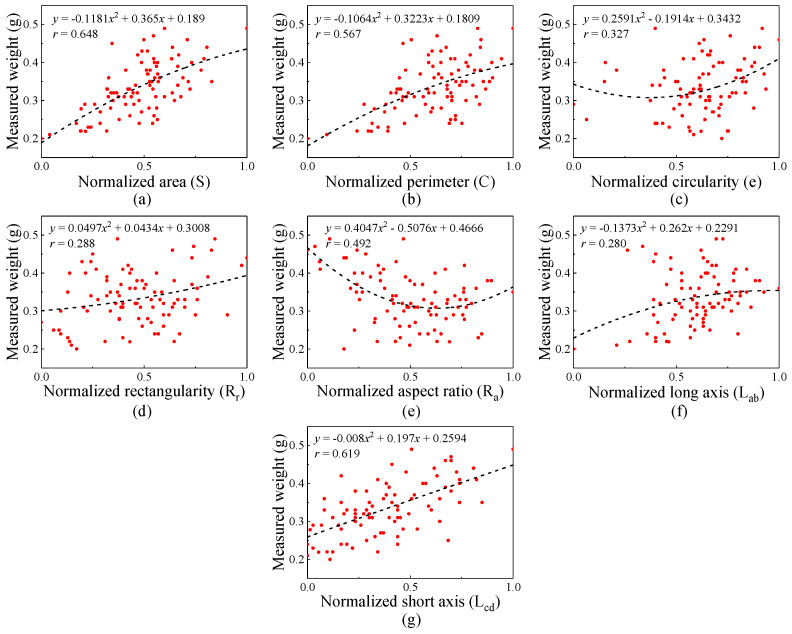
Correlation analysis between feature and weight for the unbroken kernel.

The quadratic polynomial was adopted to illustrate the correlation between individual features and the weight of unbroken kernel, which was ranked as area (*S*) > short axis (*L_cd_*) > perimeter (*C*) > aspect ratio (*R_a_*) > circularity (*e*) > rectangularity (*R_r_*) > long axis (*L_ab_*), as shown in Figure 8. The correlation between *S* and unbroken kernel weight was the highest (*r* = 0.648) because the projected area was more closely related to the unbroken kernel volume. In addition, the external features of the unbroken kernel were similar, and the kernel thickness was different. These factors result in a poor linear relationship (low *r*-value) between individual features (except area) and kernel weight.

The results of correlation analysis between maize kernel features and weight can be seen; a linear relationship exists between the maize kernel’s (broken and unbroken) external features and weight. However, the linearity between characteristics and grain weight needs to be improved. Therefore, the machine learning algorithm was used to optimize multiple kernel features to achieve accurate kernel weight prediction.

### 3.2. Performances of Weight Estimation Models Based on Machine Learning Algorithm

#### 3.2.1. Determination of Optimal Algorithm for Prediction Model

**Figure 9:**
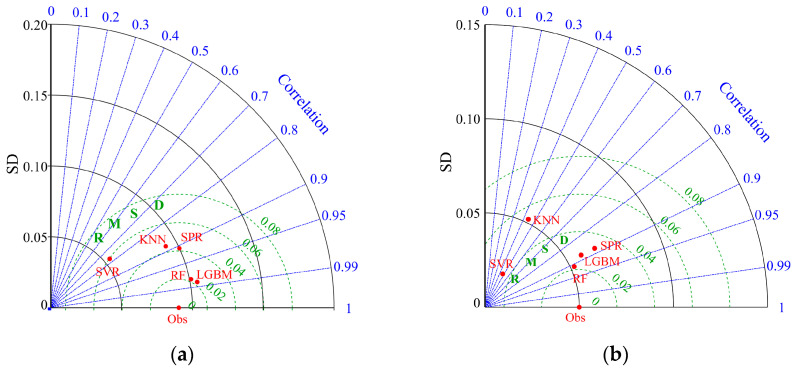
Accuracy comparison of prediction models for broken and unbroken kernel weight: (**a**) broken; (**b**) unbroken.

The accuracy of weight prediction models for broken and unbroken kernels is shown in Table 2 and Figure 9. The Taylor diagram shows kernel weight prediction models constructed by different algorithms. The advantages and disadvantages of different models can be easily evaluated using the Taylor diagram. Taylor charts help evaluate model performance by showing how model predictions compare to observations in terms of standard deviation (*SD*), correlation coefficient (*r*), and root-mean-square error (*RMSE*). In a Taylor diagram, there are usually the following elements: Observation point (Obs) represents the position of the standard deviation and mean of the observed data; radius represents the size of the standard deviation (*SD*). The farther away from the center point of the model, the greater the standard deviation. The angle between the model marker point and the horizontal line indicates the correlation (*r*) between the model and the reference data. The closer the angle is to the direction of the reference data, the stronger the correlation between the model and the observed data. The root-mean-square error (*RMSE*) is represented by marking the distance from the point to the horizontal axis observation point. The smaller the distance, the smaller the root-mean-square error of the model prediction; that is, the more accurate the model prediction.

In a word, label points of the model (marked in red), radiation (marked in blue), horizontal (vertical) axis (marked in black), and dashed lines (marked in green) in the Taylor chart represent the model, correlation coefficient (*r*), standard deviation (*SD*), and root-mean-square error (*RMSE*), respectively (Figure 8).

The comparison of prediction models for broken kernel weight is shown in Figure 9a. The *r* of the prediction model of the LGBM algorithm was 0.985 and higher than that of other algorithms. The results showed that the error was small, and the degree of linear correlation between variables was high. At the same time, the *SD* and *RMSE* of the broken kernel weight prediction model based on the LGBM algorithm were smaller than those of the other algorithms (SPR, RF, SVR, and KNN), indicating that the model has better prediction stability and higher prediction accuracy.

The comparison of prediction models for unbroken kernel weight is shown in Figure 9b. The *r* of the RF algorithm prediction model was 0.91, which was higher than that of SPR, RF, SVR, and KNN, indicating that the linear correlation between variables of the RF algorithm complete kernel weight prediction model was very high. The prediction model *r* of the SVR and KNN algorithm was much lower than others, which was unsuitable for constructing the unbroken kernel weight prediction model. In addition, the *SD* and *RMSE* of the RF weight prediction model were smaller than those of the prediction model (SPR and LightGBM), indicating that the accuracy of the RF algorithm in predicting unbroken kernel weight was higher than that of the other two algorithms. There were many differences in the geometric and shape features of broken kernels, but there were also a few differences among unbroken kernels. The broken and unbroken kernels belong to two completely different sample sets. Therefore, the optimal prediction algorithm of broken kernel weight was different from that of unbroken kernel weight prediction.

The results show that LGBM and RF algorithms are more suitable for constructing weight prediction models of broken and unbroken grains, respectively. Some scholars have also constructed weight prediction models for unbroken and broken corn kernels using deep learning algorithms. For example, Zhang et al. developed the weight prediction model of corn kernel using convolutional neural network algorithms [39]. The *r*-values of the regression models for broken and unbroken kernels were 0.996 and 0.979, respectively. Compared to deep learning algorithms, the regression models for kernel weight constructed using machine learning algorithms exhibited slightly lower *r*-values; however, they still exceeded 0.9, meeting the expected requirements. Machine learning algorithms typically have faster training speeds and lower resource demands than deep learning [40]. That allows for the more efficient and rapid construction of broken rate prediction models for various mainstream domestic corn varieties. In China, there are over 2000 registered corn varieties. Among them, the mainstream varieties for cultivation number around several dozen to a hundred [41,42]. Fast and efficient modeling methods are essential for the development of the industry. Therefore, under the condition of meeting expected requirements, machine learning algorithms may be more suitable for building similar models.

**Figure 10:**
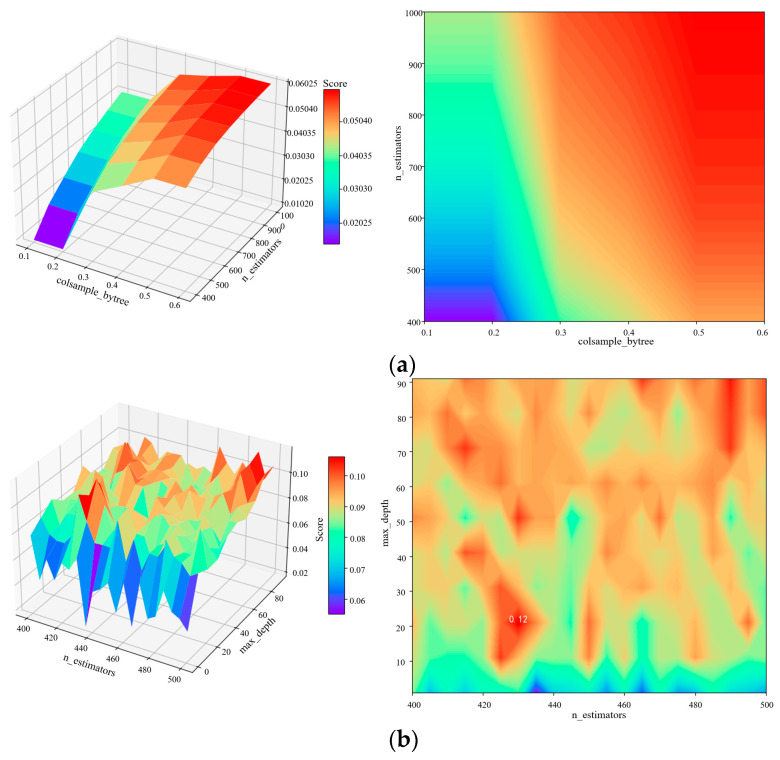
Optimization results of machine learning algorithm hyperparameters: (**a**) LGBM algorithm cross-validation results; (**b**) RF algorithm cross-validation results.

At the same time, taking the optimal machine learning algorithm as an example, the results of hyperparameter optimization are described in this manuscript. The LGBM and RF hyperparameters were optimized using the root-mean-square error of cross-validation as the evaluation index. As shown in Figure 10, the cross-validation root-mean-square error of the kernel weight prediction model constructed by LGBM and RF algorithms fluctuates significantly under different hyperparameter combinations. As shown in Figure 10a, the root-mean-square error of cross-validation was the lowest when the hyperparameter colsample_bytree ranged from 0.1 to 0.2, and n_estimators ranged from 400 to 500. This indicates that the LGBM algorithm has an optimal combination of hyperparameters in this region. As shown in Figure 10b, the *RMSE* of RF algorithm cross-validation was the lowest when the number of decision trees (n_estimators) ranged from 420 to 440, and max_depth ranged from 0 to 10. That indicates that there is an optimal combination of hyperparameters in this region.

After considering the kernel weight prediction model’s evaluation indexes (*r*, *SD*, and *RMSE*), the LGBM algorithm was more suitable for the broken kernel weight prediction model. The LGBM algorithm optimizes the combination of hyperparameters: optimal hyperparameter combination: n_jobs = −1, colsample_bytree = 0.1, n_estimators = 410, random_state = 2018. The RF algorithm was ideal for the unbroken kernel weight prediction model. The RF algorithm optimizes the combination of hyperparameters: n_estimators = 435, max_features = 5, max_depth = 6, min_samples_split = 2. The above research provides a theoretical reference for the construction of a maize kernel quality detection model.

#### 3.2.2. Comparative Analysis of Predictive Model Performance Based on Optimal Algorithm

**Figure 11:**
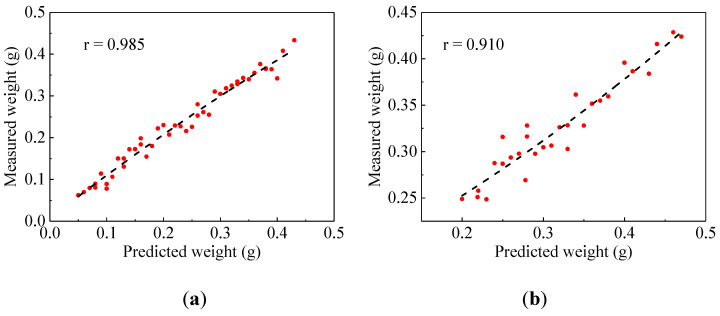
Linear relationship between predicted weight and actual weight based on the optimal algorithm: (**a**) Prediction of broken kernel weight based on LGBM algorithm; (**b**) prediction of unbroken kernel weight based on RF algorithm.

Based on the above results, it can be seen that the weight prediction performance of multiple features combined optimization was better than that of only a single feature. The LGBM algorithm was used to jointly optimize the seven features of the broken kernel (Figure 11a)). Compared with the broken kernel weight prediction by a single feature (Figure 6 and Figure 7), the linearity between the measured weight (actual weight) and the predicted weight was significantly improved by the combined prediction of seven features (*r* = 0.985). Similarly, the RF algorithm was used to jointly optimize unbroken kernels’ geometric and shape features (Figure 11b). The results showed that the linearity between the predicted weight and the measured weight of the unbroken kernel was good. The unbroken kernel weight prediction model with multiple features was better than the weight prediction model with a single feature. In addition, the *r*-value of the unbroken kernel weight prediction model was lower than that of the broken kernel weight prediction model. The high similarity of unbroken kernel characteristics easily causes the weight prediction error [21]. There are more compelling features of broken kernels, which can better predict the weight of broken kernels.

Machine learning algorithms were employed to successfully process multiple maize kernel characteristics to detect broken rates. However, there are some limitations to the machine learning algorithms. When there is a large difference between maize varieties, it is necessary to optimize the machine learning algorithm’s hyperparameters to advance the model’s accuracy. The process of adjusting hyperparameters takes experience and time, which may affect the performance of the final model. At the same time, feature selection and extraction are crucial to model performance, often requiring expert knowledge and many experiments.

### 3.3. Performance of Classification Model for Kernel Defect Detection

**Figure 12:**
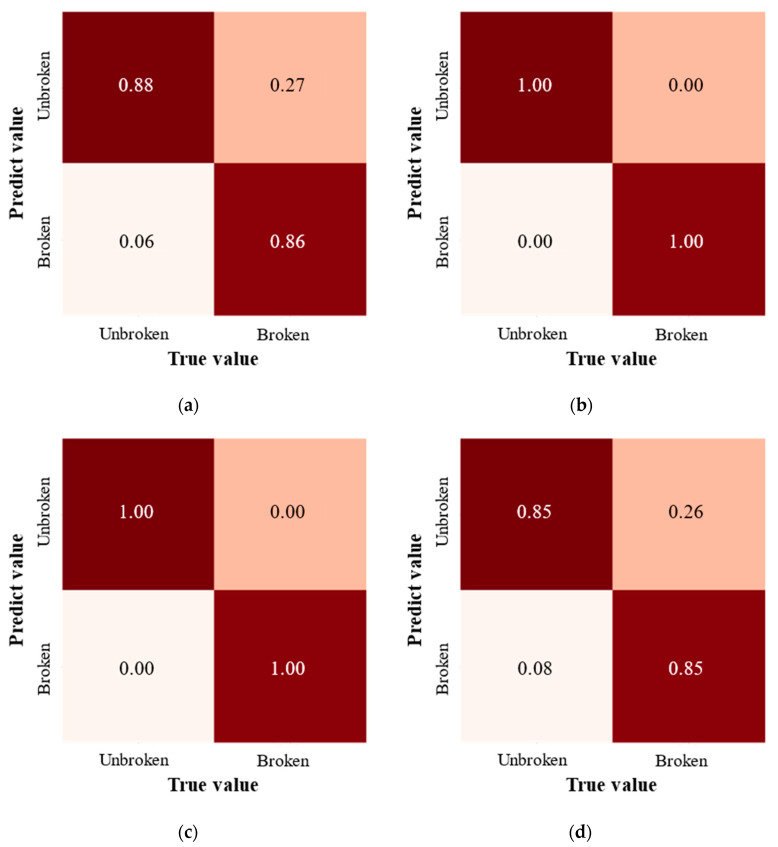
Confusion matrix of different algorithm test sets: (**a**) LGBM test set confusion matrix; (**b**) RF test set confusion matrix; (**c**) SVM test set confusion matrix; (**d**) KNN test set confusion matrix.

As shown in Figure 12, the recognition and recall rates of the unbroken and broken kernel defect detection model based on the LGBM algorithm were 0.88 and 0.86, respectively. The misjudgment rate of the broken kernel was 0.27. The recognition and recall rate of the unbroken and broken kernels in RF and SVM models was 1.0. And there was no misjudgment of the broken kernel. The recognition and recall rate of the kernel defect detection model based on the KNN algorithm was 0.85 for the unbroken kernel and broken kernel. The misjudgment rate of the broken kernel was 0.26. The experimental results showed that the target detection model of the RF and SVM algorithm has a good effect on the recognition of broken and unbroken kernels. LGBM and KNN target detection models were insufficient for unbroken and broken kernel recognition. The recall rate of unbroken kernel recognition was higher than that of broken kernel. The recognition rate of broken kernels was low because of missing detection or confusion with the unbroken kernels.

The main reason for the low recognition rate was that the boundary between individual broken kernels and unbroken kernels was sometimes challenging to define. Because the external damage feature of the kernel includes micro-cracks, crushing, coloboma, etc., compared with other features, microcracks are not easily observed [43]. The kernel shape features with tiny cracks or only slight breakage are similar to the entire maize kernel size, which presents a challenge for machine vision detection of broken kernels [44]. The detection algorithm is an essential factor affecting the recognition accuracy of machine vision. LGBM and KNN algorithms misjudge slightly broken kernels as whole kernels. Both RF and SVM algorithms have an excellent effect on the kernel recognition of fine cracks. The accuracy of kernel defect recognition models with different algorithms is shown in Table 3. The accuracy of the broken kernel recognition model constructed by the SVM algorithm was 1.0, which was better than that of the RF algorithm (model recognition accuracy was 0.947). The results are similar to those of Zhang et al. [45]. They constructed a classification model for broken and unbroken corn kernels using the YOLOv8n deep learning algorithm, achieving an accuracy of 99.1%. For the binary classification of unbroken and broken kernels, the SVM algorithm reached an accuracy of 100%. This indicates that under specific conditions, particularly when the data volume is small, features are significant, or the model is simple, machine learning algorithms can also demonstrate excellent accuracy compared to deep learning algorithms [46,47]. Therefore, the SVM algorithm was more suitable for constructing the kernel defect detection model (broken and unbroken kernel).

**Figure 13:**
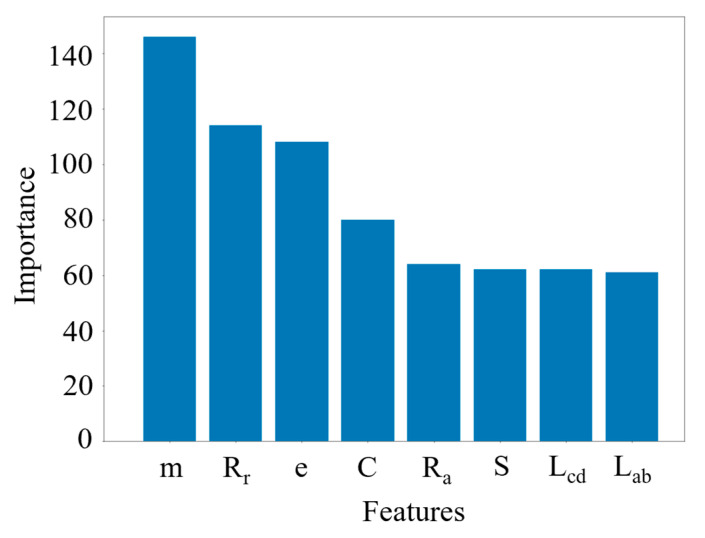
Ranking the importance of different characteristics on kernel classification.

As shown in Figure 13, the peak value of the histogram of geometric features (area, perimeter, aspect ratio, long axis, and short axis) and shape features (circularity and rectangularity) was not zero. The results showed that it influenced the recognition of broken and unbroken kernels. It was proved that the features selected in this study were meaningful for recognizing maize kernel shape features. At the same time, weight, rectangularity, circularity, and perimeter significantly influenced the recognition of broken kernels. The main reason was that the threshing machine would hit the maize kernels during the maize threshing process. The main form of kernel breakage was a lack of biological tissue, which resulted in a significantly smaller kernel weight. At the same time, the kernel lacks biological organization, considerably changing its shape and affecting features such as rectangularity, roundness, and perimeter.

Although machine learning is excellent at recognizing maize broken kernels, they cannot often make logical reasoning. When the characteristics of research objects are complex, deep learning algorithms are needed to solve them. However, these shortcomings do not mean that machine learning is not valuable; instead, they remind us to consider its applicability and potential risks when using machine learning. Therefore, the sample set can be expanded later to improve the algorithm and the classification model.

### 3.4. Analysis of Model Accuracy Difference Based on Different Algorithms

In regression prediction applications, LGBM (LightGBM) and RF (random forest) models typically demonstrate better accuracy than SPR (sparse projection regression), SVR (support vector regression), and KNN (K-nearest neighbors) algorithms, mainly due to differences in their algorithmic structure and topology. LGBM is a tree-based gradient boosting algorithm that employs a stage-wise splitting strategy, which enables high efficiency in handling large-scale datasets and allows it to capture complex features effectively [48]. In contrast, RF constructs an ensemble of multiple decision trees, reducing the risk of overfitting and increasing the model’s robustness. Moreover, both LGBM and RF can effectively model interactions between features, whereas SVR relies on setting an appropriate kernel function, and KNN only considers local neighborhoods, which lack global information. Additionally, while SPR performs well with sparse data, its ability to capture nonlinear relationships is relatively weak [49]. Therefore, LGBM and RF are more capable of identifying underlying patterns in complex data structures, leading to higher prediction accuracy.

In binary classification models, the accuracy of the SVM (support vector machine) algorithm often surpasses that of LightGBM (light gradient boosting machine), RF (random forest), and KNN (K-nearest neighbors) algorithms, primarily due to differences in algorithm structure and topology [50]. SVM works by maximizing the margin between data points and the decision boundary, which allows it to effectively handle high-dimensional data and establish clear decision boundaries, enhancing classification performance. Additionally, SVM’s kernel trick enables it to find nonlinear decision boundaries in complex feature spaces, offering flexibility in modeling complex relationships. In contrast, while LightGBM and RF can capture complex interactions among features, they may overfit on intricate patterns, especially in smaller datasets, adversely affecting accuracy. KNN relies on local neighborhood data, making it sensitive to noise and imbalanced samples, which can lead to degraded performance in complex scenarios [51]. Therefore, SVM tends to maintain higher accuracy and robustness when dealing with complex binary classification tasks.

### 3.5. Analysis of Performance Verification Test for Broken Rate Model

**Figure 14:**
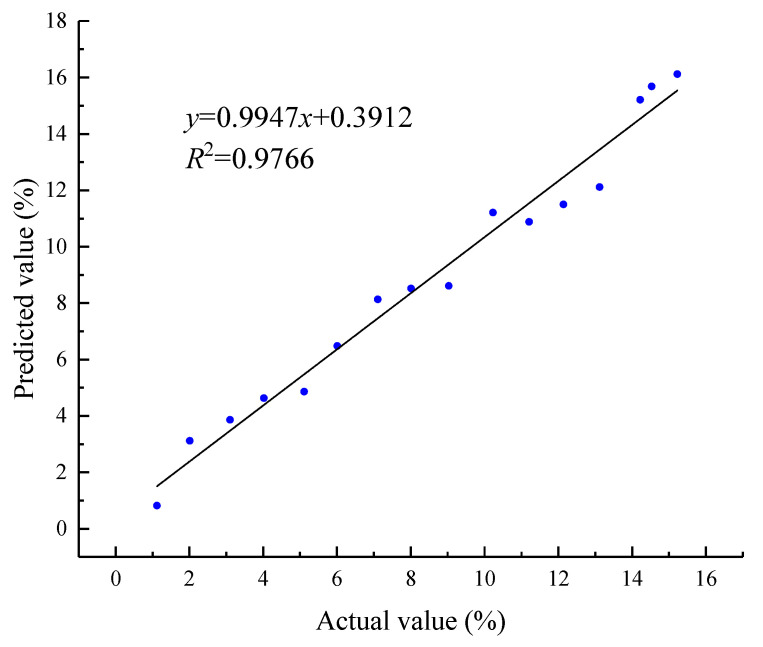
Predictive performance of maize breakage rate model.

As illustrated in Figure 14, a strong linear relationship was observed between the predicted and actual broken rates, with an *R*^2^ value of 0.9766. The experimental findings indicate that the broken rate detection model developed in this study was statistically significant for accurately detecting broken rates. In other words, the model can realize the quantitative detection of maize broken rate, offering a valuable tool for reducing harvest losses.

The broken rate detection model often has a large number of parameters and computational complexity to improve the detection accuracy as much as possible [45,52]. It requires high hardware computing resources. However, the edge computing terminal memory and computing power of the combine are low to ensure reliability in complex field environments. This results in the inference speed of complex models being limited and unable to meet the requirements of real-time broken rate detection [53]. Therefore, it is necessary to develop a lightweight detection model suitable for mobile embedded devices to detect maize broken rates in real time.

The machine learning algorithms were used to construct the maize broken rate detection model in this manuscript. Machine learning algorithms require smaller data sets to achieve good results. The need for computing resources is low and can be run on ordinary computers [54]. The detection model based on a machine learning algorithm is more suitable for real-time detection of maize kernel breakage rate. Some scholars also use deep learning algorithms to improve the accuracy of the grain broken rate model [55]. However, deep learning algorithms require large amounts of data and high computing resources. At the same time, the long training time of the model makes it difficult to realize real-time detection [56]. In this manuscript, seven characteristics of maize kernel were selected to detect the broken rate, and the data scale was small. The machine learning algorithm is more efficient in processing the data set, and the model also obtains good detection performance (*R*^2^ = 0.9766).

Secondly, this manuscript realizes the quantitative detection of corn kernel crushing rate. In this paper, a machine learning algorithm was used to construct a corn kernel classification and regression model, which realized the recognition and mass proportion of broken kernels. This method is a more scientific and quantitative description of the corn kernel breakage rate. At the same time, it also improves the speed of model detection. Kayabasi Ahmet et al. used an ANN neural network and Bayesian regularization learning algorithm to build a grain defect recognition model to achieve effective grain quality classification [57]. Although the accuracy of the classification model of crushed grain was improved by using a deep learning algorithm, the quantitative detection of grain was not realized. At present, there is little research on the quantitative online detection algorithm of corn crushing rate, and more research objects are wheat and soybean [58].

The method proposed in this paper also has important guiding significance for the construction of broken rate detection models for other maize varieties. The maize variety selected in this paper was Yichen 369. The Yichen 369 maize variety is one of the typical planting varieties in Northeast China (one of China’s main corn-producing areas). The high yield of this variety is in line with China’s current strategic demand to increase grain production. Therefore, this article chooses variety as the research object. In this manuscript, the broken rate was measured by extracting the geometric characteristics of maize kernel. The method proposed in this manuscript can be applied to other maize varieties. Due to the different sample sets, the model hyperparameters may be different when constructing the optimal broken rate detection model. This phenomenon is normal when building models based on machine learning algorithms. Therefore, the method proposed in this paper has important guiding significance for the construction of broken rate detection models for other maize varieties.

Concurrently, deep learning algorithms have broad application prospects in agricultural production [59]. The authors will further use deep learning algorithms to improve the accuracy and practicality of the detection model for maize broken rate. Moreover, to ensure the adaptability of the detection model, it is essential to expand the sample range in subsequent research.

## 4. Conclusions

In this manuscript, the broken rate prediction model was constructed to realize maize kernel quality real-time detection based on machine vision and machine learning algorithms. The weight regression and defect classification models were constructed by extracting seven shape features of maize kernel to calculate the broken rate. At the same time, five machine learning algorithms were used to optimize the model’s accuracy for better performance. The results show that the LGBM algorithm was more suitable for the broken kernel weight prediction model, with *r*, *SD*, and *RMSE* of 0.985, 0.105, and 0.022, respectively. The RF algorithm was ideal for constructing the unbroken kernel weight prediction model with *r*, *SD*, and *RMSE* of 0.910, 0.052, and 0.022, respectively. In addition, the weight, rectangularity, roundness, and perimeter significantly influenced the recognition of broken kernels. The method proposed in this study has a vital application in maize kernel defect detection, which can help in making more objective kernel inspections and improve inspection efficiencies.

## Figures and Tables

**Table 1:** Geometric feature and shape feature parameters of maize kernel.

Area	Perimeter	Aspect Ratio	Long Axis	Short Axis	Circularity	Rectangularity
S	C	R_a_ = L_ab_/L_cd_	L_ab_	*L_cd_*	e = S/S_2_	R_r_ = S/S_1_

**Table 2:** Precision comparison of different models.

Model	Prediction of Broken Kernel Weight			Prediction of Unbroken Kernel Weight		
	*r*	*SD*	*RMSE*	*r*	*SD*	*RMSE*
SPR	0.908	0.100	0.042	0.881	0.066	0.032
LightGBM	0.985	0.105	0.022	0.879	0.058	0.028
RF	0.98	0.101	0.022	0.910	0.052	0.022
SVR	0.77	0.054	0.060	0.470	0.020	0.044
KNN	0.882	0.092	0.044	0.442	0.052	0.054

**Table 3:** Accuracy of broken and unbroken kernel recognition models.

Algorithm	LightGBM	RF	SVM	KNN
Accuracy	0.867	0.947	1.000	0.853

## Data Availability

The original contributions presented in the study are included in the article, further inquiries can be directed to the corresponding author.
